# Dr. Ciro de Quadros, Global immunization advocate and leader

**DOI:** 10.11604/pamj.2014.18.103.4713

**Published:** 2014-05-29

**Authors:** Robin Biellik

**Affiliations:** 1 Consultant Epidemiologist, Geneva, Switzerland

**Keywords:** Ciro de Quadros, epidemiology, polio eradication, public health, leader

## Obituary

Dr. Ciro de Quadros, global immunization advocate and leader, passed away quietly in Washington DC last night, May 28, 2014. He fought a long battle with cancer. For those of you who, like myself, had the privilege to work directly with Ciro, I am sure you share with me the sense of loss of this inspiring public health leader. For those of you who did not know him, Ciro is, to a large extent, the reason why immunization has been such a resounding public health success. Inspired by his experience of working on smallpox eradication (much of it in the Horn of Africa), and energized by the success of polio elimination under his leadership in the Region of the Americas, he became a forceful advocate and dedicated supporter of measles and then rubella elimination. Most of the elimination strategies and we follow today were proposed, tested and proven in the Americas under Ciro's direction.

Ciro was born in the state of Rio Grande do Sul in Brazil in 1940. Ciro was at the fore-front of global efforts to control, eliminate or eradicate vaccine-preventable diseases for nearly 4 decades, most of that time as Director of the Expanded Programme on Immunization (EPI) at the Pan-American Health Organization (PAHO). In 2003, Ciro joined the Sabin Vaccine Institute, a non-profit organization honoring the legacy of Albert Sabin, developer of the oral polio vaccine (OPV).

Ciro was also a keen advocate for the introduction of new vaccines, e.g. rotavirus, rubella, human papilloma virus, pneumococcal and others, and on issues related to the sustainability of national immunization programs. He was also a member of the faculty at Johns Hopkins School of Hygiene and Public Health and the School of Medicine of the George Washington University. He has been deservedly awarded many honours and prizes available (except maybe the Nobel Peace Prize), and has accepted them all with his characteristic humour and humility.

All that I can realistically add is a few words from the perspective of a European vaccine-preventable disease (VPD) epidemiologist who worked for him. I had that privilege for 4 years from 1987 to 1991.

When I was in my 2nd year at CDC as an Epidemic Intelligence Service (EIS) Officer with the then Division of Immunization, Ciro requested a Spanish-speaking epidemiologist for a special mission. PAHO had introduced the use of an indicator of surveillance sensitivity for suspected polio (acute flaccid paralysis, or AFP) and, at that time, the minimum performance expected from all countries was to find at least 1 case of AFP per 100,000 population (the indicator has since been refined). However, Mexico and Central America claimed that it was impossible to find so many cases so the indicator was not broadly applicable. Ciro sent me to Mexico and Central America to undertake active case searches where I found hundreds of unreported AFP cases, proving that the indicator was indeed valid. When I graduated from EIS, Ciro offered me a job, which I gladly accepted. Ciro hired me as Inter-Country EPI Advisor for the "Southern Cone" (Argentina, Brazil, Chile, Paraguay and Uruguay) based at the PAHO's office in Brasilia, Brazil. The next 4 years were inspirational. I learned as much or more from the public health experts at the Brazilian Ministry of Health and State Health Departments than they learned from me. Thanks to Ciro, I learned many innovations in EPI including the computerization and electronic transmission of VPD surveillance data, how to plan and implement national immunization days (NIDs), and effective mechanisms for strengthening routine immunization service delivery. I also had the privilege to assist in a study to optimize the global formulation of OPV. The last case of polio occurred on my watch in 1989 in northeast Brazil, and the first steps were taken on the road to eliminate measles.

Ciro invested deeply in his staff and molded them into a family, to which we were very proud to belong. In public, he always defended his staff in a most professional manner. In private, he might express dissatisfaction if someone had not implemented the work to his high standards, but any rebuke would remain strictly out of the public view. In this way, morale among the staff always remained very high.

Ciro's legacy will remain with us all for decades to come, as we continue to work with diligence and enthusiasm towards his vision, that is, a World free of vaccine-preventable diseases.

**Figure:**
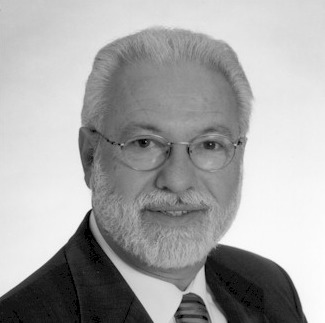
Ciro de Quadros (Photo Wikipedia)

